# Repurposing Drugs for Acute Myeloid Leukemia: A Worthy Cause or a Futile Pursuit?

**DOI:** 10.3390/cancers12020441

**Published:** 2020-02-13

**Authors:** Anna V. Wojcicki, Meena Kadapakkam, Adam Frymoyer, Norman Lacayo, Hee-Don Chae, Kathleen M. Sakamoto

**Affiliations:** 1 Division of Hematology/Oncology, Department of Pediatrics, Stanford University School of Medicine, Stanford, CA 94305, USA; avwoj@stanford.edu (A.V.W.); mkadapak@stanford.edu (M.K.); lacayon@stanford.edu (N.L.); heedon.chae@gmail.com (H.-D.C.); 2 Division of Neonatal and Developmental Medicine, Department of Pediatrics, Stanford University School of Medicine, Stanford, CA 94305, USA; frymoyer@stanford.edu

**Keywords:** Acute myeloid leukemia, drug repurposing, drug development, targeted therapies, mechanism of action

## Abstract

Acute myeloid leukemia (AML) is a clinically and genetically heterogenous malignancy of myeloid progenitor cells that affects patients of all ages. Despite decades of research and improvement in overall outcomes, standard therapy remains ineffective for certain subtypes of AML. Current treatment is intensive and leads to a number of secondary effects with varying results by patient population. Due to the high cost of discovery and an unmet need for new targeted therapies that are well tolerated, alternative drug development strategies have become increasingly attractive. Repurposing existing drugs is one approach to identify new therapies with fewer financial and regulatory hurdles. In this review, we provide an overview of previously U.S. Food and Drug Administration (FDA) approved non-chemotherapy drugs under investigation for the treatment of AML.

## 1. Introduction

Drug repurposing is the practice of finding new applications for existing drugs outside their initial approval. The concept of repurposing began fortuitously with drugs such as sildenafil, first tested as an antihypertensive drug, eventually becoming a successful treatment for erectile dysfunction [1]. Compared to traditional drug development, drug repurposing is a time- and cost-effective alternative [1,2].

Traditional drug development costs an average of 2.5 billion dollars from discovery to market [3]. Although success rates have improved due to the use of biomarkers and the emergence of immunotherapy, the success rate of oncology trials remains a dismal 3.4% [4]. Over 50% of clinical trials fail due to inadequate efficacy, 17% due to safety concerns and 22% because of lack of funding [5]. One advantage of repurposing is that drugs in clinical use typically have a known safety and toxicity profile. Although repurposed drugs still need to undergo clinical testing for a new indication, the development timeline can be accelerated with less setbacks and lower cost than traditional drug development.

Oncology is a field in need of effective and low-cost treatments [6]. Tapping into the pool of known and approved drugs presents an attractive avenue for developing new therapies. Acute myeloid leukemia (AML) is characterized by unrestricted proliferation of myeloid progenitor cells. A buildup of chromosome aberrations, gene fusion products and mutations in regulators of hematopoietic growth and differentiation leads to the accumulation of immature abnormal blasts [7]. Despite progress in understanding the pathophysiology of AML, standard induction chemotherapy consisting of a 7-day infusion of cytarabine in conjunction with anthracycline on days 1-3 remains largely unchanged since it was first described in the 1970s [8,9].

AML affects patients of all ages and poor prognosis is associated with older age and complex cytogenetics. Even for those who respond favorably to treatment, relapse is common and leads to poor outcomes. Forty five percent of patients with AML will experience relapse of which only 11% will survive to 5 years [10]. Survivors of AML often experience secondary late effects resulting from chemotherapy and allogeneic hematopoietic cell transplantation (HCT) including cardiotoxicity, infertility, secondary neoplasms, central nervous system dysfunction and decreased quality of life [11]. High demand exists for new therapies that are more effective, less toxic and better positioned to target the critical molecular pathways in AML leukemogenesis.

**Figure 1:**
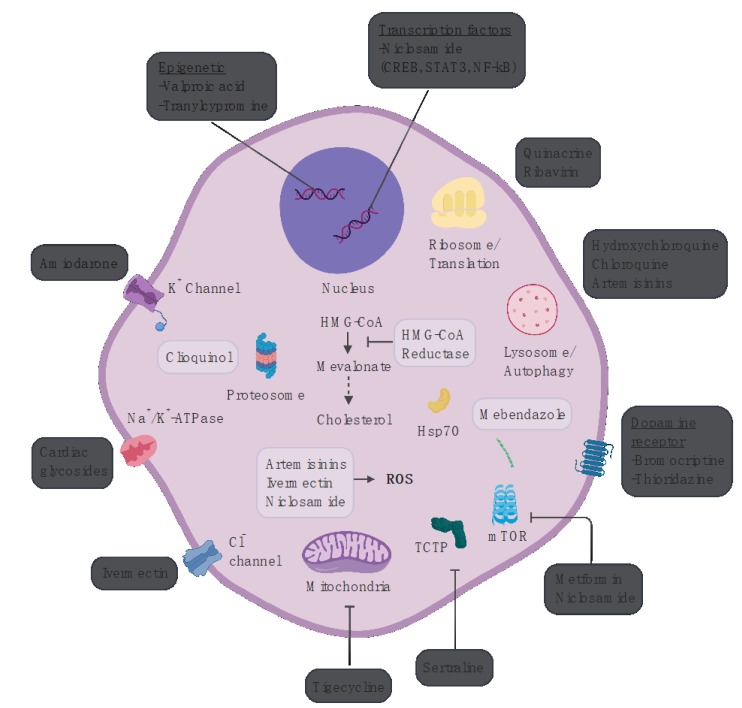
Schematic representation of cellular targets for AML repurposing candidates. For compounds whose activity against AML is not well defined the approved mechanism of action is depicted. Abbreviations: CREB: cAMP Response Element Binding protein; STAT3: signal transducer and activator of transcription 3; NF-kB: nuclear factor kappa-light-chain-enhancer of activated B cells; HMG-CoA: enzyme 3-hydroxy-3-methyl-glutaryl-CoA; ROS: reactive oxygen species; TCTP: translationally controlled tumor protein; mTOR: mammalian target of rapamycin. Figure created with Biorender.com.

In the last five years, the development of targeted therapies for AML has started to accelerate. U.S. Food and Drug Administration (FDA) approved therapeutics, CD33 immunoconjugate gemtuzumab ozogamicin, fms like tyrosine kinase 3 (FLT3) inhibitor midostaurin, and isocitrate dehydrogenase 2 (IDH2) inhibitor enasidenib [12], all address specific features or subtypes of AML. These drugs provide credence to using AML disease pathogenesis to inform the development of compounds with activity against precise molecular aberrations. Repurposed drugs represent an additional source of agents with potential to augment existing therapies. Characteristics of ideal repurposing candidates include well defined targets, differential toxicity between leukemia cells and normal cells in vitro and favorable pharmacology. Many agents are identified by high throughput screens of cancer cell lines, observational studies, in silico screens and bioinformatic approaches. In this review, we will survey the landscape of non-chemotherapeutic drugs with potential for repurposing in AML (Figure 1).

## 2. Repurposing for AML

### 2.1. Antimicrobials

#### 2.1.1. Anthelmintic

Niclosamide is an FDA-approved oral agent used to treat tapeworm infections [13]. As an anthelmintic, niclosamide exerts its effects through disrupting oxidative phosphorylation and targeting the mitochondria, although this mechanism of action has not been well elucidated [14]. Niclosamide has been shown to inhibit a number of signaling pathways including nuclear factor kappa-light-chain-enhancer of activated B cells (NF-kB) [15], mammalian target of rapamycin complex 1 (mTORC1) [16], signal transducer and activator of transcription 3 (STAT3) [17], and Wnt/B-catenin [18] in several cancer cells. While niclosamide has been reported to inhibit the NF-kB pathway and generate reactive oxygen species (ROS) to induce apoptosis in AML cells [15], niclosamide was found to share structural similarity with a small molecule inhibitor of cAMP Response Element Binding protein (CREB) and suppress the proliferation of AML cells by inhibiting CREB-dependent signaling pathways [19]. CREB is overexpressed in AML and associated with poor prognosis [20,21]. Niclosamide prolonged survival of AML patient derived xenograft (PDX) mice and pretreatment with niclosamide potentiates the effect of chemotherapeutics [19]. Niclosamide acts synergistically with chemotherapy such as cytarabine and daunorubicin, making this drug an ideal candidate for early-phase clinical trials.

Mebendazole is commonly used to treat intestinal helminthiasis by binding irreversibly to tubulin and blocking microtubule assembly [22]. However, in models of AML, mebendazole causes disruption of the heat shock protein 70 chaperon system, leading to the degradation of transcription factor c-MYB in AML cells [23]. Mebendazole was identified as a c-MYB targeting drug by using a gene expression signature of *MLL*-rearranged AML to probe the Connectivity Map database. Mebendazole inhibited the viability and colony forming activity of *MLL* rearranged and non-rearranged human cell lines in vitro and prolonged the survival of *MLL*-*AF9* xenograft mice [23]. Both mebendazole and niclosamide possess good safety profiles as evidenced by their decades of use in the developing world, making them favorable candidates for the treatment of AML. Although no clinical trials have investigated the activity of mebendazole against AML, six studies evaluating mebendazole as a treatment for cancers including colorectal and glioma are registered with the National Institutes of Health’s (NIH) Clinicaltrials.Gov [24].

Clioquinol was developed as an oral antiparasitic for treating amebiasis but in the United States, it is primarily used to treat dermatologic disorders. Clioquinol works as a proteasome inhibitor through a copper-dependent mechanism in AML and other hematologic malignancies [25]. Furthermore, serum copper levels are elevated in hematological malignancy patients in relapse or progressive disease [26], which may contribute to clioquinol’s preferential toxicity for malignant cells. A phase I study of clioquinol in patients with refractory AML, myelodysplastic syndrome (MDS), acute lymphocytic leukemia (ALL) and chronic lymphocytic leukemia (CLL) determined its safety and efficacy [27] (Table 1). Clioquinol had minimal effect on proteasome activity and no clinical response was observed. Although plasma levels of clioquinol were within target range, intracellular levels of clioquinol were low, varied and did not correlate with plasma levels [27]. Further investigation is needed to better understand clioquinol’s metabolism and devise strategies for increasing cytosolic concentration.

Another antiparasitic, ivermectin, suppresses the cellular viability of multiple AML cells and patient samples in vitro. In vivo, ivermectin delayed the progression of human and mouse leukemia in 3 models of AML. Ivermectin’s mechanism of action as an antiparasitic is related to the activation of glutamate gated chloride channels in invertebrates [28]. Similarly, caspase-dependent apoptosis in AML cells is induced by ivermectin through chloride-dependent membrane hyperpolarization and ROS generation. Ivermectin showed a synergistic effect with cytarabine and daunorubicin [29]. Although preclinical results have been promising, concerns about limited differential sensitivity between AML and normal hematopoietic cells [29] have likely kept ivermectin from clinical trials.

#### 2.1.2. Antibacterial

Leukemia cells utilize mitochondrial translation and oxidative phosphorylation to a higher degree than hematopoietic stem cells [30]. Tigecycline is an antimicrobial agent with activity against Gram-positive and Gram-negative bacteria [31]. Tigecycline selectively killed leukemic stem and progenitor cells by inhibiting mitochondrial translation [32]. In xenograft mouse models, tigecycline delayed disease progression with comparable potency to daunorubicin and bortezomib at maximally tolerated dose. Selective killing of AML cells while sparing healthy cells makes tigecycline an attractive potential therapeutic. A phase I clinical trial evaluating the tolerance and biologic activity of intravenous infusions of tigecycline in relapsed or refractory AML patients was completed in January of 2015 (NCT01332786). As one-hour infusions of tigecycline were insufficient to maintain a steady state level of drug due to a short half-life [33], no clinical response was observed in the 27 patients treated with 42 cycles of tigecycline over seven doses. Currently, the approved formulation of tigecycline in unstable after reconstitution. Developing a stable formulation of tigecycline that allows for continuous intravenous infusion may be crucial to achieving a sufficient steady-state concentration and clinical efficacy.

#### 2.1.3. Antiprotozoal

The artemisinins (ARTs) are a family of antimalarial drugs derived from the plant *Artemisia annua*. ARTs exhibited anti-leukemic effects and demonstrated a synergistic effect with chemotherapeutics in AML cells in vitro [34] through the generation of ROS, caspase activation, decreased lysosomal integrity [34], and activation of p38 mitogen-activated protein kinase [35]. ARTs are intriguing candidates for repurposing because of their wide use on a global scale and sustained record of safety [36].

Antimalarial agents hydroxychloroquine and chloroquine are under investigation in clinical trials for a variety of cancers including glioblastoma multiforme, pancreatic adenocarcinoma, sarcoma, multiple myeloma, melanoma and AML [37]. Hydroxychloroquine induced cell death by inhibiting autophagic degradation in AML cells [38]. Autophagy is a self-degradative intracellular process implicated in both tumor suppression and promotion [39]. Cell death induced by hydroxychloroquine was higher in cytarabine-resistant leukemia cell lines compared to cytarabine-sensitive cells. Furthermore, treatment of cytarabine- resistant cells with hydroxychloroquine increased their sensitivity to cytarabine. While combination therapy of hydroxychloroquine with mitoxantrone and etoposide was investigated in a phase I clinical trial for relapsed AML, the trial was terminated due to slow accrual (NCT02631252).

The antimalarial quinacrine has also received interest as a candidate for repurposing in AML. In a screen of 1266 compounds from the LOPAC^1280^ library, quinacrine was the only compound that showed high cytotoxic activity (% inhibition) against primary leukemia cells and four AML cell lines with low toxicity in normal mononuclear cells in vitro. Bioinformatic analysis of gene expression suggests that quinacrine targets ribosome biogenesis by inhibiting ribosomal DNA transcription [40]. In a follow up study, quinacrine demonstrated in vivo efficacy in AML PDX mice without toxicity by decreasing the number of tumor cells in the blood by 70% and significantly prolonging survival. The combination of cytarabine and quinacrine produced a synergistic effect in vivo and in vitro, increasing the mean survival time of treatments compared to controls by 171% [41].

#### 2.1.4. Antivirals

Ribavirin, an antiviral guanosine analogue, is a 5’ mRNA 7-methyl guanosine (m7G) cap competitor and inhibits eukaryotic translation initiation factor 4E (eIF4E) activity. eIF4E enhances the translation of proteins involved in cellular survival and proliferation through binding to the m7G cap [42]. Ribavirin suppressed colony forming activity of patient AML cells [43]. The M4/M5 subtype of acute myeloid leukemia overexpresses eukaryotic translation initiation factor eIF4E [44]. These data led to a clinical trial testing the efficacy of ribavirin monotherapy in M4/M5 AML patients with relapsed or refractory disease unable to undergo induction therapy [45]. There was one complete remission (CR) and two partial remissions without significant treatment-related toxicity out of 11 evaluable patients through the suppression monocytic blasts and decreased eIF4E protein level. Another phase I clinical trial of ribavirin in combination with low-dose cytarabine for relapsed and refractory AML was conducted in eIF4E-overexpressing patients [46]. Combination treatment resulted in 2 CRs, one partial remission and two blast responses out of 21 patients. Clinical response was correlated with reduction of *eIF4E* mRNA levels and relocalization of eIF4E protein to the cytoplasm. In an effort to understand the lack of clinical response to ribavirin, factors affecting pharmacokinetics were investigated. Interaction between eIF4E and ribavirin was inhibited by increased levels of sonic hedgehog transcription factor Gli1. Patients with markers of impaired drug uptake including low levels of the ribavirin transporter (ENT1) and adenosine kinase (ADK)—an enzyme required to metabolize ribavirin—were also resistant to treatment. Additional study is necessary for optimal clinical efficacy. A clinical trial of ribavirin and hedgehog inhibitor with or without decitabine in AML is recruiting (NCT02073838).

### 2.2. Metabolism

#### 2.2.1. HMG-CoA Reductase Inhibitors

Statins are a family of drugs that lower blood cholesterol levels by inhibiting the enzyme 3-hydroxy-3-methyl-glutaryl-CoA (HMG-CoA) reductase and blocking the conversion of HMG-CoA to mevalonic acid, the rate-limiting step of the biosynthesis of cholesterol and other isoprenoids [47]. Genes involved in the mevalonate pathway are overexpressed in AML [48]. AML cells exposed to radio-chemotherapy increase their production of cholesterol as a protective response [49]. Pretreating AML cells with a cholesterol synthesis inhibitor sensitizes the cells to subsequent chemotherapeutic treatment [49]. Meta-analysis of 14 observational studies found statin use was negatively associated with the risk of developing hematological malignancy [50]. These observations led to clinical trials testing the safety and efficacy of HMG-CoA reductase inhibitors for the treatment of AML. Escalating doses of pravastatin were co-administered with idarubicin/high dose cytarabine to block the adaptive cholesterol response in AML cells [51]. Of the 37 patients enrolled, 20 achieved CR/complete remission with incomplete platelet recovery (CRp) without toxicity associated with pravastatin [51]. In a phase II study (NCT00840177) of idarubicin and cytarabine in combination with pravastatin for relapsed AML, the response rate was 75% and treatment was well tolerated [52]. However, a study testing the efficacy of idarubicin, cytarabine, and pravastatin as induction therapy for untreated AML and high-risk MDS concluded that the regimen did not meet the criterion for efficacy (CR rate of 70%) [53]. Currently, there are two clinical trials ongoing including a phase I/II study of lovastatin with high-dose cytarabine for refractory or relapsed AML (NCT00583102) and a pilot trial of atorvastatin in tumor protein 53 mutant and p53 wild-type malignancies (NCT03560882).

#### 2.2.2. Metformin

Metformin is used for the treatment of diabetes mellitus type 2 by decreasing gluconeogenesis and increasing glycolysis and insulin sensitivity [54]. Various meta-analyses have noted a relationship between metformin use and decreased risk of cancer and cancer related mortality in patients with diabetes [55,56]. Due to the anticancer potential reported in epidemiological studies, metformin has been well researched in preclinical models. Mammalian target of rapamycin (mTOR) regulates cellular metabolism and is strongly implicated in the development of cancer and diabetes [57]. Metformin interferes with the proliferation of AML cells by inhibiting the activation of mTOR through the liver kinase B1 (LKB1)/5’ adenosine monophosphate-activated protein kinase (AMPK)/tuberous sclerosis protein (TSC) tumor suppressor axis while sparing normal hematopoiesis [58]. In an AML xenograft mouse model, metformin inhibited the growth of AML cells in vivo without evidence of toxicity [58]. Based on these encouraging results, the evaluation of metformin was pursued in a phase I clinical trial in combination with cytarabine for the treatment of relapsed or refractory AML. Unfortunately, the study was terminated due to slow accrual (NCT01849276).

### 2.3. Neuropsychiatric

#### 2.3.1. Valproic Acid

Aberrant DNA methylation and histone acetylation are key features of malignancy. Because these processes are reversible, there is interest in developing therapeutics which target cancer epigenetics [59]. Valproic acid (VPA) is a short chain fatty acid that is widely used as an anticonvulsant [60]. VPA has a number of cellular effects, including potentiation of gamma aminobutyric acid (GABA) neurotransmission and alteration in the expression of multiple genes. Most notably, VPA inhibits histone deacetylase activity [61]. Histone deacetylation inhibitors restore altered gene expression by increasing the binding of regulatory machinery such as transcription factors [62]. VPA induces differentiation of transformed cells, including AML cell lines and patient AML blasts [61].

VPA, as a therapeutic for AML and MDS, has been investigated in a number of phase I and II clinical trials with varied results. Several clinical trials of VPA for the treatment of AML have reported minimal clinical efficacy with associated toxicity [63,64]. Patients with AML secondary to MDS seem to be more responsive to VPA therapy. In a study of VPA to treat 58 patients with AML, response rates were 5% for patients using AML criteria and 16% for patients using MDS criteria with no significant difference between VPA monotherapy and all-trans retinoic acid (ATRA) combination therapy [65]. Similarly, VPA appears to have a better clinical response in patients with low risk MDS (52% response rate) when compared to AML patients (16%) [66]. In a phase II study of 5-azacitidine, VPA and ATRA combination treatment of high-risk AML and MDS patients, the response rate was 26% including 22% CR [67]. There was no evidence for blast differentiation by VPA treatment. Interestingly, demethylation of genes involved in the progression of MDS to AML (FZD9, ALOX12, HPN, and CALCA) was related to clinical response.

However, VPA did not improve clinical output in patients with MDS or elderly patients with AML in a phase II randomized study of low-dose decitabine with VPA [68]. This finding was validated in a randomized phase II trial of 204 patients comparing decitabine +/− VPA +/− ATRA in newly diagnosed elderly AML patients. Addition of VPA did not affect the objective response rate or overall survival [69]. A randomized phase III study assessing the safety and efficacy of combining VPA and ATRA with induction therapy (idarubicin and cytarabine) for the treatment of elderly AML patients was terminated early due to lack of clinical improvement in the VPA group compared to standard treatment [70]. Hematologic toxicity and higher death rates due to VPA were thought to contribute to the poor outcomes in the group. However, relapse free survival was significantly increased in the VPA group (24.4% vs 6.4%) and patients with the NPM1 mutation had significantly superior relapse free survival and overall survival after treatment with VPA in comparison to the standard group [70]. VPA may not be superior to standard therapy but could be useful in treating certain types of AML such as AML secondary to MDS and NPM1 positive AML.

#### 2.3.2. Ergot Alkaloid/Dopamine

Bromocriptine is used to treat Parkinson’s disease as an agonist at the D2 dopamine receptor and an antagonist at the D4 dopamine receptor [71]. In an effort to identify less toxic therapies for elderly adults with MDS and secondary AML, a gene expression dataset of 229 MDS and 224 AML patients was used to develop gene-based signatures for the progression of MDS. These signatures were coupled to a database of compounds and used to identify novel therapeutics by connectivity mapping. Bromocriptine was one of 4 compounds identified for screening. Treatment of AML and high-risk MDS cell lines with bromocriptine reduced cell viability, increased apoptosis and induced myeloid differentiation with little effect on normal cells [72]. Furthermore, a synergistic effect was observed when AML cells were treated with cytarabine and bromocriptine in vitro [73]. The mechanism of action of bromocriptine remains unknown. Other dopamine agonists were tested in a drug screen and determined ineffective, suggesting bromocriptine’s antiproliferative properties are unrelated to dopamine receptors [73]. To date, bromocriptine has not been investigated in animal models or clinical trials for AML.

#### 2.3.3. Thioridazine

Thioridazine (TDZ) is a first-generation phenothiazine antipsychotic used to treat schizophrenia as a dopamine receptor antagonist. Due to severe cardiovascular side effects, including QT interval prolongation, TDZ was withdrawn from the market in 2005 [74]. In vitro testing of TDZ in neoplastic human pluripotent stem cells selectively induced differentiation and reduced AML clonogenic growth without affecting normal human pluripotent stem cells [75]. This work led to a phase I clinical trial to evaluate the safety of TDZ in combination with cytarabine in patients with relapsed or refractory AML. Eight of the 11 patients experienced a reduction in blast levels after TDZ monotherapy [76]. Reduction was associated with the expression of the dopamine receptor D2 on leukemia cells prior to initiation of therapy. Dose limiting toxicity was reported in 1 patient who experienced grade 3 QTc interval prolongation. Due to these effects, plasma levels of TDZ were not able to rise to the level that demonstrated efficacy in vitro.

#### 2.3.4. Tranylcypromine

The lysine residues of histone tails represent key areas of epigenetic modification leading to either increases or decreases in gene expression depending on the type and position of DNA methylation [77]. Lysine-specific demethylase 1A (LSD1) reverses the process of DNA methylation by removing methyl groups from the H3K9me1/2 histone and other non-histone substrates such as p53 and E2F1 [78,79,80]. Loss of LSD1 impairs differentiation of hematopoietic stem cells and causes pancytopenia in mice [81]. LSD1 is required for *MLL*-*AF9* cells to initiate AML and knockdown or inhibition of LSD1 suppresses the clonogenic and repopulating potential of AML leukemic stem cells (LSCs) [82,83]. Antidepressant tranylcypromine (TCP), a monoamine oxidase inhibitor, inhibits LSD1 activity. Based on the theory that ATRA therapy is ineffective in non-APL AML due to a block in the transcriptional activation of retinoic acid receptor target genes, researchers tested the hypothesis that treatment with a LSD1 inhibitor could “unlock” ATRA’s effect. Treatment of ATRA-insensitive exhausted T (TEX) cells with ATRA and TCP increased myeloid differentiation [84]. ATRA treatment with TCP significantly diminished the engraftment of leukemic cells compared to untreated mice [84]. Identification of LSD1 as a therapeutic target led to the initiation of several phase I clinical trials evaluating the safety and efficacy of TCP in combination with ATRA (NCT02717884, NCT02273102, NCT02261779). Due to its limited potency and selectivity, efforts have been made to develop derivatives of TCP with clinical efficacy [82,85,86]. 

#### 2.3.5. Sertraline

The selective serotonin reuptake inhibitor antidepressant sertraline is the most commonly prescribed psychiatric medication among adults [87]. Translationally controlled tumor protein (TCTP) is involved in cell cycle progression and its expression levels are controlled by extracellular signals. TCTP induces histamine release as an extracellular protein, which protects cells from apoptosis [88]. TCTP is significantly downregulated in tumor cells that have reverted to a nonmalignant phenotype [89]. It was postulated that compounds inhibiting the histaminic pathway could inhibit the expression of TCTP. Sertraline was tested due to its structural similarity with other antihistaminic drugs and identified as a compound with the ability to downregulate TCTP and cause cytotoxicity in the U937 leukemia cell line [90]. Sertraline inhibited proliferation of AML cells by inducing apoptosis and autophagy [91]. Currently, a phase I trial evaluating sertraline and cytosine arabinoside in adults with relapsed and refractory AML is recruiting (NCT02891278).

### 2.4. Antiarrhythmic

#### 2.4.1. Cardiac Glycosides

The cardiac glycosides (CGDs) are a family of drugs used to treat congestive heart failure and arrhythmia. By inhibiting the Na^+^/K^+^-ATPase pump, CGDs enhance the amount of intracellular Ca^2+^ causing a positive inotropic effect [92]. Digitoxin inhibits growth and induces apoptosis in human malignant cell lines without affecting highly proliferating normal cells. Patients taking digitoxin for cardiac disease have a lower incidence of leukemia/lymphoma and kidney/urinary tract cancers compared to the general population, suggesting an association between increased plasma concentration of digitoxin and decreased risk of those cancers [93]. CGDs decreased the viability of AML LSCs in vitro at a greater sensitivity than normal hematopoietic stem cells [94]. Despite promising results of anti-cancer activity in vitro, CGDs suppress general protein synthesis through Na^+^/K^+^-ATPase pump inhibition to exert cytotoxicity, an effect that is not specific for cancer cells. Furthermore, mouse cells are resistant to CGDs and can tolerate high plasma levels of CGDs, distorting the translatability of mouse xenograft models to humans [95]. To date, one phase Ib/II study of the safety and activity of digoxin with decitabine in adult AML and MDS was opened but eventually terminated due to slow accrual (NCT03113071).

#### 2.4.2. Amiodarone

Amiodarone is a class III antiarrhythmic that lengthens the cardiac action potential through blocking potassium channels. Amiodarone was identified as a compound possessing synergy with the Bcl-2 inhibitor ABT-263 [96]. Bcl-2 is overexpressed in AML cells resistant to chemotherapy and associated with poor prognosis [97]. Treatment of AML cell lines with a combination of amiodarone and ABT-263 enhanced apoptosis through reduction of AKT phosphorylation and activation of caspase-3 [96]. Experiments are needed to confirm the in vivo efficacy and safety of amiodarone.

## 3. Conclusions

Repurposing drugs is one strategy for pursuing effective and potentially less toxic therapies to treat the diverse and heterogeneous array of hematologic malignancies. Repurposing drugs that are already FDA approved represents “low hanging fruit” and is worth pursuing when sound preclinical data exists to support the efficacy and safety of the particular drug. To date, there have not been any “home runs” with regards to repurposing drugs to treat AML. Valproic acid, ribavirin and HMG-CoA reductase inhibitors have been tested in clinical trials with varying degrees of success. These compounds have well-defined targets and a wealth of preclinical data and observational studies to support their investigation in clinical trials. Although researchers estimate that over 75% of drugs have the potential to be repurposed, the number is closer to 6% for drugs tested in a different therapeutic area from their original indication [98]. For the many drugs that have stalled in phase I and II clinical trials, the issue is often about questions of efficacy rather than safety concerns.

Lack of efficacy in clinical trials is the primary hurdle for drug repurposing in AML (Table 2). Ribavirin, tigecycline, HMG-CoA reductase inhibitors, clioquinol and VPA all demonstrated robust activity against AML preclinically in vitro and in vivo with little success in clinical trials. Pharmacokinetics was the main concern for trials of clioquinol, ribavirin and tigecycline. Theoretically, optimizing drug formulation and dosing could lead to clinical trial success in these particular cases. However, failure of preclinical models to accurately recapitulate AML remains an area of concern overall.

For diseases where animal models do not sufficiently capture the human phenotype, preclinical data are not always predictive of patient response [99]. Even earlier in the process, in vitro models struggle to represent the biological complexity and heterogeneity of malignancy. Candidates are chosen for their cytotoxic effect against primary cells and AML cell lines through measures of decreased viability, increased apoptosis and decreased proliferation. However, these outputs are meaningless if the model is not characteristic of AML. Leukemia relapse occurs due to the inability of therapies to target LSCs. Several repurposing studies attempted to assess the activity of an agent against LSCs by utilizing cell lines with properties similar to LSCs. For example, Harris et al used *MLL*-*AF9* AML colony-forming cells because formation of blast-like colonies correlates with LSC potential [82]. Technologies such as 3-D organoids are emerging as models better able to preserve the character of tumor cells and their microenvironment [100]. While simple to use and low cost, 2D culture systems that are not translatable result in the long-term cost of failed clinical trials due to lack of efficacy. Another popular solution is in silico screening [2]. Through leveraging big data, researchers are able to identify potential therapeutics at a relatively low cost before using resources to conduct in vitro and in vivo studies. 

AML is a disease of many subtypes with varying outcomes. There are several different types of chromosome abnormalities and genetic mutations of a number of genes including *FLT3*, *TP53*, *NPM1*, *CEBPA*, and *RUNX1*. These abnormalities are associated with prognosis and response to therapy [8]. Although cytarabine and anthracycline are the standard chemotherapy drugs used to treat AML, the heterogenous nature of this disease makes it impervious to “one stop shop” types of treatment. Targeted treatments which address specific mutations and pathways are key to addressing the development and proliferation of AML. As with standard therapy, there will always be patients that do not respond to targeted treatment. To increase the success rates of repurposing, Nowak-Sliwinska et al. propose a personalized approach that combines pharmacologic, genetic, and clinical data to predict patients who are more likely to respond with informed phenotypic assays in the preclinical phases of development [101]. Efforts to improve efficacy will also need to address the design of preclinical model systems to account for the heterogeneity of AML. This will require collecting and integrating data from multiple sources including primary cell culture, animal models and distinct patient populations.

Though drug repurposing is a more economically feasible approach to drug development- in practice this is not always the case. Bringing a drug from discovery to market requires a significant monetary investment, even if the cost appears lower at face value compared to a traditional approach. The upside of novel drug development is that a guarantee of market exclusivity exists at the end of the road. Candidates for repurposing are submitted through FDA application 505(b)(2) and Article 10 of the European Medicines Agency (EMA). In the United States, varying degrees of market exclusivity are granted based on status (orphan drug, new chemical entity, pediatric) [102]. Unfortunately, repurposed drugs face challenges in obtaining patent protection, especially when the compound is already being prescribed off label or if acceptance of the drug for a different indication already exists in the public domain [1]. Drugs with new formulations are eligible for new composition of matter patents making them the most attractive repurposing opportunity for commercial success [2]. Effective repurposing requires alternative sources of funding and unique collaborations between pharmaceutical companies, motivated academic researchers and philanthropic organizations. Funding sources for drug repurposing initiatives are available through the National Centre for Advancing Translational Sciences and a number of philanthropic organizations including Cures within Reach, Findacure and the Anticancer Fund [2]. For drugs such as metformin, hydroxychloroquine and cardiac glycosides, whose clinical trials were terminated due to slow accrual, extra funding may be the key to trial success.

Even drugs that fail repurposing attempts may provide valuable insight about the mechanism of AML. The antipsychotic tranylcypromine, for example, sparked the development of derivatives with better selectivity and potency, a strategy which could be pursued with other compounds that show promise but fall short in terms of efficacy. On the other hand, a drug like valproic acid may be better positioned to treat patients with specific mutations (NPM1) or subtypes of AML (AML secondary to MDS). In all likelihood repurposed drugs will not achieve success as comprehensive agents. Rather, attention should be given to identifying specific mutations integral to the various subtypes of AML and using repurposed drugs to augment the pool of potential therapies. An integrated approach that pairs traditional drug discovery with repurposing and utilizes a number of modalities including genetic association studies, retrospective clinical analysis, high throughput screening technology, and computational analysis should be leveraged to improve the prospects of patients with AML.

## Figures and Tables

**Table 1:** Repurposed clinical candidates for AML *.

Drug	Original Indication	Clinical Trials
Tigecycline	Community acquired pneumonia, Intra-abdominal infections, Skin infections	Safety study evaluating intravenous infusions of tigecycline to treat acute myeloid leukemia (NCT01332786)
Hydroxychloroquine	Lupus erythematosus, Malaria, Rheumatoid arthritis	-Phase I study of mitoxantrone & etoposide combined w/hydroxychloroquine, for relapsed acute myelogenous leukemia (NCT02631252)
Clioquinol	Dermatologic disorders	-Study evaluating the tolerance and biological activity of oral clioquinol in patients w/relapsed or refractory hematologic malignancy (NCT00963495)
Ribavirin	Hepatitis C	-Use of ribavirin & low dose Ara-C to treat acute myeloid leukemia (NCT01056523) -Ribavirin & hedgehog inhibitor with or without decitabine in AML (NCT02073838) -A study of ribavirin to treat M4 and M5 acute myelocytic leukemia (NCT00559091) -Study of decitabine in combination with sequential rapamycin or ribavirin in high risk AML patients (NCT02109744)
Atorvastatin	Hypercholesterolemia, prevention of coronary artery disease	-A pilot trial of atorvastatin in tumor protein 53 (p53)- mutant and p53 wild-type malignancies (NCT03560882)
Lovastatin	Hypercholesterolemia, prevention of coronary artery disease	-Dose escalation phase I/II study of lovastatin w/high dose cytarabine for refractory or relapsed AML (NCT00583102)
Pravastatin	Dysbetalipoproteinemia, hypercholesterolemia, cardiovascular disease prevention	-Idarubicin, cytarabine and pravastatin sodium in treating patients w/ acute myeloid leukemia or MDS (NCT01831232) -Cyclosporine, pravastatin sodium, etoposide and mitoxantrone hydrochloride in treating patients w/relapsed or refractory AML (NCT01342887) -S0919 idarubicin, cytarabine, and pravastatin in treating patients w/ relapsed AML (NCT00840177)
Metformin	Diabetes mellitus, type 2	-Metformin + cytarabine for the treatment of relapsed/refractory AML (NCT01849276)
Valproic acid	Bipolar disorder, epilepsy, migraine prophylaxis	-Study of decitabine alone or in combination w/valproic acid and all-trans retinoic acid in acute myeloid leukemia (NCT00867672) -Decitabine w/ or w/o valproic acid in MDS & AML (NCT00414310) -Differentiation induction in AML (NCT00175812) -Vidaza & valproic acid post allogeneic transplant for high risk AML and MDS (NCT02124174) -Treatment of acute leukemia relapse after allotransplantation (NCT01369368) -Disease stabilization in AML by treatment w/ATRA, valproic acid & low dose cytarabine (NCT00995332) -Azacytidine w/valproic acid versus Ara-C in AML/MDS patients (NCT00382590) -5-azacytidine valproic acid and ATRA in AML and high risk MDS (NCT00339196) -Phase II 5-azacytidine plus VPA plus ATRA (NCT00326170) -Decitabine & valproic acid in treating patients w/refractory or relapsed AML or previously treated CLL or SLL (NCT00079378)
Thioridazine	Schizophrenia	-Safety study of thioridazine in combination w/cytarabine to treat relapsed or refractory AML (NCT02096289)
Tranylcypromine	Major depressive disorder	-Study of sensitization of non-M3 AML blasts to ATRA by epigenetic treatment w/tranylcypromine (NCT02717884) -Phase I study of TCP-ATRA for adult patients w/AML and MDS (NCT02273102) -Phase I/II trial of ATRA and TCP in patients w/relapsed or refractory AML & no intensive treatment is possible (NCT02261779)
Sertraline	Major depressive disorder, OCD, panic disorder, PTSD, PMDD, Social anxiety disorder	-Sertraline and cytosine arabinoside in adults w/relapsed and refractory AML (NCT02891278)
Digoxin	Atrial fibrillation and flutter, heart failure	-Safety and activity of digoxin w/decitabine in adult AML and MDS (NCT03113071)

* Acute myeloid leukemia (AML), myelodysplastic syndrome (MDS), valproic acid (VPA), all-trans retinoic acid (ATRA), chronic lymphocytic leukemia (CLL), small lymphocytic lymphoma (SLL), tranylcypromine (TCP), obsessive compulsive disorder (OCD), post-traumatic stress disorder (PTSD), premenstrual dysphoric disorder (PMDD).

**Table 2:** Outcomes of repurposing trials for AML as reported by ClinicalTrials.gov.

Lack of Efficacy	Toxicity	Slow Accrual	Ongoing
-Clioquinol -Ribavirin -HMG-CoA Reductase Inhibitors -Tigecycline -Valproic Acid	-Thioridazine	-Cardiac glycosides -Hydroxychloroquine -Metformin	-Tranylcypromine (3) -Sertraline -Ribavirin (2) -HMG-CoA Reductase Inhibitors (2) -Valproic acid (2)
